# Environmental Risk Assessment and Confinement of Genetically Engineered Trees with an Emphasis on Vegetative Reproduction

**DOI:** 10.3390/plants15162496

**Published:** 2026-08-18

**Authors:** Yongil Yang, C. Neal Stewart

**Affiliations:** 1 Center for Agricultural Synthetic Biology, University of Tennessee Institute of Agriculture, Knoxville, TN 37996, USA; 2 Department of Plant Sciences, University of Tennessee, Knoxville, TN 37996, USA

**Keywords:** gene editing, vegetative reproduction, risk assessment, gene flow, genetic engineered tree, confinement of asexual reproduction, field test

## Abstract

Genetic engineering (GE) and gene editing may endow traits to trees such as increased biomass and the production of novel biomaterials. Long-lived organisms such as trees might be subject to biotechnology-related risks that could be different than those of annual row crops. Those risks could be relevant to production in engineered plantations and beyond plantations to natural forests. Therefore, appropriate risk regulation is important to assure biosafety of commercialized engineered trees. In addition to gene flow via sexual reproduction, vegetative reproduction might play an additional role in environmental “exposure” risk relative to transgene dispersal in GE tree plantations. While vegetative reproduction is beneficial for preserving desired genetic traits during tree propagation, it may lead to proximal clonal spread in the field. Although the environmental risks associated with vegetative reproduction of GE trees are recognized in commercial forestry, there are few field-based environmental risk assessment (ERA) studies on dispersal risks of self-propagated GE trees. GE or gene editing of target genes involved in the vegetative propagation processes may be useful to mitigate environmental risks of clonal spread through vegetative reproduction. This review provides updates for recent field test results of GE and gene edited trees. Gene candidates related to vegetative reproduction including adventitious shooting (AS) and adventitious rooting (AR) are discussed herein as a means to mitigate unintended clonal spread from GE tree plantations.

## 1. Introduction

Genetic engineering (GE) of woody plants, especially trees, is being performed to alter biomass production, among other traits. There are concerns that long-lived plants such as trees could have unique environmental risks over the centuries or even millennia of their lives. Recent technical advances, using gene editing and RNA interference without stable gene integration, may help mitigate risks by minimizing the amount of new DNA incorporated into plants. These approaches also may decrease public fears of GE. These advances facilitate the expansion of GE applications to satisfy agricultural environmental applications that cannot be addressed by classic breeding. Environmental release of GE trees is subject to varying regulations throughout the world on a country-by-country basis. While few GE trees have been commercialized for release into the environment, there are many examples of GE traits relevant to biosafety risk assessment, which is the basis of this review paper.

This review discusses the environmental risk assessment of GE and gene edited trees and related biosafety concerns. We will explore gene or clonal spread scenarios from both sexual and vegetative propagation of trees.

## 2. Use of GE Trees in Forestry

Trees have vast ecological and economic roles: biodiversity, regulation of water resources and atmospheric CO_2_ levels, and the production of biomass and bioproducts [1]. However, global forest area and productivity have trended downward in recent decades, leading to losses in species biodiversity and ultimately extinctions. The recovery cost of biodiversity losses was estimated at approximately US $490 billion per year [2]. GE traits often can enhance plant fitness and can be both potentially ecologically positive or negative; risk assessment is important to understand context and relative magnitude of risks [3,4]. In particular, GE trees may play a role in increasing forest productivity but we must strive to maintain ecological sustainability.

To date, GE trees have been mainly developed for enhanced biomass production, biofuel feedstock, and for production of consumer-based chemicals or materials [5]. GE trees are typically modified by altering single or multiple genes related to biomass synthesis, tolerance to abiotic and biotic stresses, pest and herbicide resistance, and phytoremediation of contaminated soils [6,7]. For several decades, GE trees were classically generated using *Agrobacterium*-mediated transformation with gene constructs consisting of promoters, genes of interest, and transcription terminators derived from various prokaryotic and eukaryotic sources. However, this approach often impairs consistent transcript expression owing to variation in T-DNA insertion number and genomic location insertion site. Therefore, the risk assessment of GE trees has sometimes focused on mitigating inherent risk of impairment of untargeted gene function caused by random insertion of T-DNA and spread of inserted genes to other interfertile relatives in the wild.

## 3. Advances of Gene Editing Approaches for Trees

Gene editing techniques can overcome inherent limitations of conventional breeding and T-DNA based GE approaches [7]. Gene editing employing a site-directed nuclease such as clustered regulatory interspaced short palindromic repeats (CRISPR)/Cas9, transcription activator-like effector nuclease (TALENs), or zinc finger nucleases (ZFNs) have been widely utilized to generate gene editing crops to avoid random T-DNA transgene insertion into the host genome [6]. Of these, CRISPR/Cas9 has been popularly applied for DNA deletion or insertion by making double-strand DNA breaks at the targeted bases by targeting with guide RNA. The double-strand DNA breaks are then repaired by endogenous repair systems such as nonhomologous end-joining (NHEJ) and homology-directed repair (HDR) pathways [6,8,9]. However, off-targeting unintended locations in the genome is a frequent problem in CRISPR-mediated gene editing. Recently, the efficiency of CRISPR/Cas9 has been improved by altering the active domain of Cas9. The currently advanced tools using Cas9 protein variants in animals, humans, and plants have been detailed in Wang and Doudna’s publication [9]. As a variant of CRISPR-based genome editing, prime editing enables precise nucleotide substitutions and small insertions or deletions at targeted genome loci without requiring homology-directed repair (HDR) through the use of a Cas9-reverse transcriptase fusion protein [10,11]. However, few gene editing trees have been produced from HDR-mediated editing and prime editing. Most gene editing in trees has been performed using conventional CRISPR/Cas9 approaches. Nonetheless, gene editing in woody plants has been performed on a wide range of host genera: *Pinus*, *Hevea*, *Populus*, *Malus*, *Pyrus*, *Citrus*, *Parasponia*, *Coffea*, and *Theobroma* [7]. Current research also indicates that gene edited plants have no greater inherent risks than conventionally bred plants [7]. Additionally, rational application of artificial intelligence (AI) approaches may enhance and discover gene editing reagents for precise targeting and better engineering [12].

Most gene editing in plants is produced by T-DNA delivery of Cas9 reagents, which are subsequently removed by sexual breeding. However, the clean-up of T-DNA insertions remains challenging in tree taxa having delayed sexual breeding stages or those that are mainly vegetatively reproduced. To address this issue, T-DNA sequences can be removed post-transformation via recombinase approaches. Also, transient transformation strategies with T-DNA-deleting plasmids have been utilized for host-induced gene silencing [13,14] and spray-induced transient gene expression. These approaches can retain gene edited traits but eliminate unnecessary DNA [8,15]. However, long development cycles as well as delayed sexual reproduction make gene deletion challenging in trees. Despite technical advances in biotechnology, major regulatory challenges remain, particularly regarding the global trade and commercial use of gene edited plants. Nonetheless, efforts have been made to reduce gene editing regulatory barriers and facilitate commercialization of plants with new traits [16].

## 4. Revision of Regulatory Policy of Products from Plants Using Gene Editing

The United States plant biotechnology regulations date back to 1986 in which the so-called coordinated framework was implemented by adopting existing rules in the Department of Agriculture (USDA), Food and Drug Administration (FDA), and Environmental Protection Agency (EPA). These agencies continue to update policies and rules that are applied to engineered plants. Of importance were updates to include gene editing approaches. The USDA revised Code 7 of Federal Regulation (CFR) 340 in May 2020 to essentially exempt from regulation those plant products of gene editing techniques involving cellular repair of a targeted DNA without an externally provided repair template, single base pair targeting, or introducing a sequence corresponding to a known allele in the host gene pool [17]. The FDA also updated its policies to regulate food products derived from new plant varieties with stricter safety assessment through on- and off-target sequencing based on next-generation sequencing techniques (FDA 2019-D-4658) within the existing regulatory framework. Countries in the European Union (EU) have also moved to establish new regulatory frameworks to accommodate gene editing technologies in crop markets, with similar policy revisions [17,18]. Currently, the EU has established a new regulatory framework that is not based on existing legislation and regulations, unlike those of other countries such as the United Kingdom, the United States, and Canada [16,18,19]. This trend emphasizes a lead authority with strong responsibility for all types of bioproducts developed through new technologies, including gene editing [19]. Although gene edited plants have been favorably treated by many agricultural policies and regulatory frameworks, rigorous risk assessment is still required to establish sound regulatory policies [16,17,18,19,20]. To comply with these regulations, multiple field experiments of identical gene edited tree lines compared to their non-GE counterparts are indispensable to assess environmental risk of gene edited trees, which requires formidable costs. Therefore, frameworks for assessing risks in gene edited trees are critical.

## 5. Practical Risk Assessment of GE Trees in Field Tests

Environmental safety associated with GE crops is critical to widespread application of GE techniques in commercial agriculture. To date, the governments of at least 29 countries implement environmental risk assessment (ERA) processes, which generally address seven major risk assessment criteria: (1) persistence and invasiveness of the GE plant, or its compatible relatives, including plant-to-plant gene transfer; (2) plant-to-microorganism gene transfer; (3) interaction of the GE plant with target organisms; (4) interaction of the GE plant with non-target organisms; (5) impact of the specific cultivation, management, and harvesting techniques, including consideration of the production systems, and the receiving environments(s); (6) effects on biogeochemical processes; and (7) effects on human and animal health (feed and food webs) [21,22].

Because of their longer life spans and different reproductive strategies compared with herbaceous GE species, GE trees may have distinct fitness and invasiveness risks, depending on target genes/traits. For example, lignin reduction is one goal for GE tree production, which might enable improved biofuel and pulp production since lignin is a major source of recalcitrance that limits glucose release and cellulose fiber production in processed cell walls/biomass [23,24]. However, GE trees with modified lignin content often exhibit reduced growth and biomass, potentially owing to abnormal lignin biosynthesis during developmental stages.

A recent summary of 20 years of field tests involving lignin-reduced GE trees show that the extent of lignin reduction strongly depends on the specific gene(s) targeted within the lignin biosynthesis pathway [25]. GE trees with reduced expression of 4CL, CCoAOMT, CCR, or CAD showed more severe lignin reduction under growth chamber conditions than field conditions [26]. Interestingly, CCR-, CAD-, and 4CL-downregulated GE trees did not have altered lignin content under greenhouse conditions while exhibiting reduced lignin levels in confined field tests. Moreover, reduction in lignin content in 4CL- and CAD-downregulated poplar resulted in decreased growth and productivity depending on geographic location and coppicing practices of specific genotypes [27]. These results indicate that reduced lignin production is highly dependent on both environment conditions and genotype.

However, GE tree performance in field tests is not always highly variable across environments. For example, GE RNAi poplar for suppression of isoprene emission had consistently reduced isoprene synthesis among environments [26]. In this study, there was no significant differences in biomass observed in GE poplars grown for four years at two sites in Arizona and Oregon, United States [26]. These results demonstrate that water and air temperature regimes did not affect the stable expression of the targeted trait in GE poplars over multiple growing seasons. In contrast to previous lignin-modified GE trees, the engineered trait in these GE poplars remained stable across different field environments.

Taken together, GE tree performance cannot be reliably predicted based solely on observations from a single environment and in the absence of field experiments. Most of all, uncertainty regarding the persistence of novel traits in GE trees largely depends on interactions between genotype and environmental factors that synergistically influence trait expression under field conditions. Therefore, practical development of GE trees requires comparative evaluations between field and greenhouse to fully characterize engineered traits. Defining appropriate and rational parameters for field testing will be fundamental to balance industrial utility with environmental safety and risk analysis.

## 6. Gene Flow and Invasiveness Issues of GE Trees in the Field

Risks of GE plants in field tests mainly consider several factors associated with persistence and invasiveness of GE plants, including gene flow of transgenes into non-target organisms. Glyphosate-tolerant GE poplar has shown impacts on weed control and tree growth under field conditions as herbicide tolerance in GE poplar resulted in approximately 20% faster growth [28]. Invasiveness and potential gene flow from faster growing in the GE poplar was discussed in this study. Potential biosafety issues such as biological invasions, horizontal and vertical gene transfer, and the affect to surrounding plant species associated with poplar taxa have been highlighted in applicable studies [29,30].

Several key aspects are considered to assess risk mitigation of GE trees in confined field tests: the self-reproductive capacity of the host tree; the existence of any sexually compatible species in the surrounding area; long-distance dispersal of propagules; and ecological interactions with species of particular concern [29]. Endogenous traits in parent species such as reproductive capacity, long-distance dispersal, and availability for gene flow must be evaluated to determine the suitability and effectiveness of control measures. Depending on the specific genetic changes, different phenotypic effects and potential ecological impacts may vary. Therefore, evaluation of expected gene transfer and phenotypic alteration resulting from the same or similar genetic modifications in sexually compatible species growing within the intended production area is also required to address the biosafety of GE trees in the field [31,32]. Therefore, in combination with morphological and physiological observations, the collection of molecular and physiological data of GE trees and neighboring trees in confined field trials provides an effective way for problem formulation and risk assessment in support of regulatory compliance in natural environments.

Several commercial *Populus* species have been derived through natural hybridization mediated by wind pollination. The genus exhibits typical reproductive traits such as hirsute seeds that promote long-distance dispersal. These characteristics increase the potential for gene movement, which GE *Populus* present a relatively high risk of transgene flow to other *Populus* species and closely related tree taxa [29]. Also, ornamental trees have been developed successfully by artificial crossing, which implies gene flow may have both positive and negative effects in wild and artificial settings [29]. To date, transgene flow has been observed in related species grown near GE poplar, eucalyptus, and plum in tree species [33,34,35,36].

Gene flow of tree transgenes has been predicted through detection of morphologic and genetic markers of hybrid poplar plantations into a wild population of *Populus trichocarpa* after cultivation over 20 years in the Pacific Northwest region of the United States [37]. This study demonstrated that pollen and seed dispersal can be spatially extensive but the level of introgression from the hybrid plantation to surrounding areas may be extremely low. Transgene introgression was detected in a wild population located at a distance of 450 m from GE trees via pollen flow. In other studies, hybrid poplar pollen effectively reached other trees as far as 7.6 km [38]; however, gene introgression of transgenes into wild *Populus* populations was as low as ~2% of the population, even at close proximity and after long (years) cultivation [39,40].

Other GE tree species, such as plum (*Prunus domestica*), have been shown to generally be of low risk as determined by experiments in the field in West Virginia, United States, over eleven years of cultivation [34]. An experimental ecosystem model was established in a 0.46 ha field planted with sentinel plum, mixed plum varieties, and honeybee hives (*Apis mellifera*) at distances surrounding GE plum trees expressing a GUS marker gene. Gene flow from pollen dispersed from GE plum to non-GE plums was low, i.e., 0.31% of the 12,116 seeds over a total of 11 years. Gene flow decreased by distance from the GE plum trees: 0 to 400 m, which was similar to that observed in poplar plantations [37]. Furthermore, plum transgene flow was identified in a single year among the total 11 years of the experiment. This study showed that the distance separating GE from non-GE trees is an important factor in gene flow, which is a consistent observation with the field poplar results [34,37].

There is concern that certain transgenes could be riskier in some hosts than others, which has spurred research on biocontainment. The sterility of pollen or embryos have been engineered by altering the regulation of expression of flowering-related genes such as LEAFY, LEAFY ORTHOLOG, AGAMOUS, APETALA1, SHORT VEGETATIVE PHASE, FLOWERING LOCUS T, and SHORT VEGETATIVE PHASE-LIKE genes in the *Eucalyptus* and *Populus* genera [33,41,42]. Field tests have been performed to assess gene flow of flowering-altered GE trees, which indicated that these genes were useful to reduce gene flow [7,32,33,43,44].

GE poplar with altered expression of fifteen different MADS family genes was studied in a single 3.6 ha field trial over seven growing seasons. Plants had floral sterility and reduced flowering traits. The three different female GE poplar hybrid clones tested had no observable vegetative growth cost [42]. Seeds collected during the experiments were sterile and did not germinate in field conditions, implying that this strategy effectively prevents gene flow.

Seeds produced by many wild-type tree species exhibit low germination rates under natural conditions. Therefore, whether the GE applications to enhance seed sterility are an effective strategy to control seedling dispersal is debated. For example, seedling survival of GE poplar was different in lab vs. field conditions with no survival of seedlings in the field [42]. Gene flow of GE *Populus nigra* containing a *Bacillus thuringiensis* (Bt) insect-resistance gene was detected in surrounding poplars located at 500 m from GE poplars at a frequency of 0.02%, but it was not detected beyond that distance. Seeds of the GE poplars could germinate, but seedlings did not survive longer than three weeks under field conditions. Interestingly, after cleaning and trimming, watering, weeding, and maintaining high humidity, 1.7% of collected seeds germinated and survived for eight weeks. These results indicate that gene flow from GE trees can occur at an extremely low level and can be managed by physical treatments [45]. Poplars can spread via clonal propagation in the field. In a field study of poplar seed sprouting or root suckers proximal to a GE plantation, low numbers of vegetative shoots from root suckers were observed but no seed-derived seedlings were observed [42,46].

## 7. Risk Assessment of Commercialized GE Trees

Given that China allowed the commercialization of several insect-resistant *Populus* varieties earlier than most other countries, there are abundant physiological and molecular data from commercial field plantations over a long period. Insect-resistant GE poplars (*P. nigra* and various poplar hybrids) were developed through *Agrobacterium*-mediated T-DNA transformation using either exogenous *Bacillus thuringiensis* (Bt) genes alone or in combination with *Sagittaria sagittifolia* API gene [47]. To date, two insect-resistant GE poplar species have been approved for commercialization. Furthermore, several commercialized transgenic poplar varieties containing the Bt Cry1Ac gene have been monitored continuously in field plantations over the long term, which enables year-by-year assessment of transgene persistence and ecological safety [48,49]. For instance, the GE poplar variety 107 carrying Bt Cry1AC gene was evaluated for the persistence of the Bt transgenes at genomic, transcript, and protein levels, together with analysis of insect resistance from 2015 to 2024 [48]. The study found no evidence of either integration or loss of the Bt gene on the chromosome, and no disruption of the Bt gene expression was observed. These results indicate that the GE poplar sustained the insect-resistance trait without molecular-level alterations under commercial field conditions for up to 10 years. Most importantly, this study demonstrated that GE poplars exhibited faster growth as a result of reduced insect damage (with an inhibition rate exceeding 65%) and required less chemical pesticide use, which would reduce pest control costs in commercial GE poplar plantations [48].

Another study investigated the ecological safety of five-year-old insect-resistant GE poplar with Bt Cry1Ac and Cry3A genes via analyses of soil properties, gene flow, and soil microbial diversity under unconfined field conditions [49]. The GE poplar also effectively inhibited pest attacks in this study. The results indicated that no transgene transfer occurred from the GE poplar to non-target organisms such as soil microorganisms. Furthermore, no negative effects on microbial biodiversity were observed during the five-year study period.

Brazil approved the commercial release of biomass-improved GE *Eucalyptus* H421, which overexpressed the Arabidopsis 1,4-β-endoglucanase Cel1 gene, following six years of risk assessment in 2015. Leaf metabolites as well as gene flow through pollen and distant seed dispersal were monitored. No significant difference in oil content or chemical composition was observed between GE and non-GE eucalyptus trees. In addition, transgene flow was detected at a rate of 1.5% through crossing with surrounding trees located near the GE eucalyptus plantation; however, no seedling establishment was observed [50,51,52]. A report on the continuous risk assessment of GE eucalyptus following commercial release was published recently [53]. According to this study, GE eucalyptus H421 has not presented any safety concerns since its commercial approval.

Although the United States has developed various GE trees to date, their commercialization has been delayed owing to ongoing debate regarding environmental concerns, regulatory frameworks, and public perception. For example, blight-tolerant American chestnut has been developed through *Agrobacterium*-mediated T-DNA transformation of wheat oxalate oxidase-encoding gene [54]. The field test of this GE tree is still undergoing regulatory review. Virus-resistant GE plum and papaya were also developed earlier and subsequently approved for commercial cultivation [55,56,57]. However, GE plum has not yet been commercially marketed.

GE orchard trees have increasingly been developed for commercialization. Viral disease-resistant GE papaya and non-brown transgenic apple have been deregulated and approved for commercial sale as human food or animal feed in China, the United States, and Canada [57,58,59]. While GE-specific hazards can be nebulous, there are remaining concerns about the waste and residual GE woody biomass after fruit harvest, highlighting the need for continued monitoring [56,60].

The establishment of biosafety paradigms for ERA of commercialized GE trees requires long-term evaluation. Although the concerns and risk assessment paradigms differ between unconfined and confined field trials and unconfined releases, the development of effective biosafety frameworks depends on precise information collected from confined field trials prior to commercialization. Gene editing techniques can substantially reduce the development period for GE trees.

To date, sixteen countries have approved field trials of GE and gene edited trees obtaining useful traits such as disease resistance, fruit production, biomass increase, carbon sequestration, and climate change retardation. The objectives and conditions of these field tests conducted in different countries have been comprehensively reviewed in recent publications [56,60]. Consequently, the number of commercialized GE trees is expected to increase significantly in the future, driven by favorable socioeconomic benefits and evidence of relatively low environmental risk.

## 8. Risk Assessment for Vegetative Reproduction of GE Trees

Trees may vegetatively reproduce by stump sprouting, air layers, and root suckers. In the U.S. Pacific Northwest, it was found that in poplar, vegetative propagule dispersal was a significant contributing factor to spread in wild field conditions, while long-distance seed dispersal occurred primarily under commercial cultivation conditions [37]. Although the potential environmental risks associated with vegetative reproduction have been discussed in several field experiments involving GE trees, practical approaches for risk assessment of vegetative reproduction remain limited [32,37,42,45,46].

**Figure 1:**
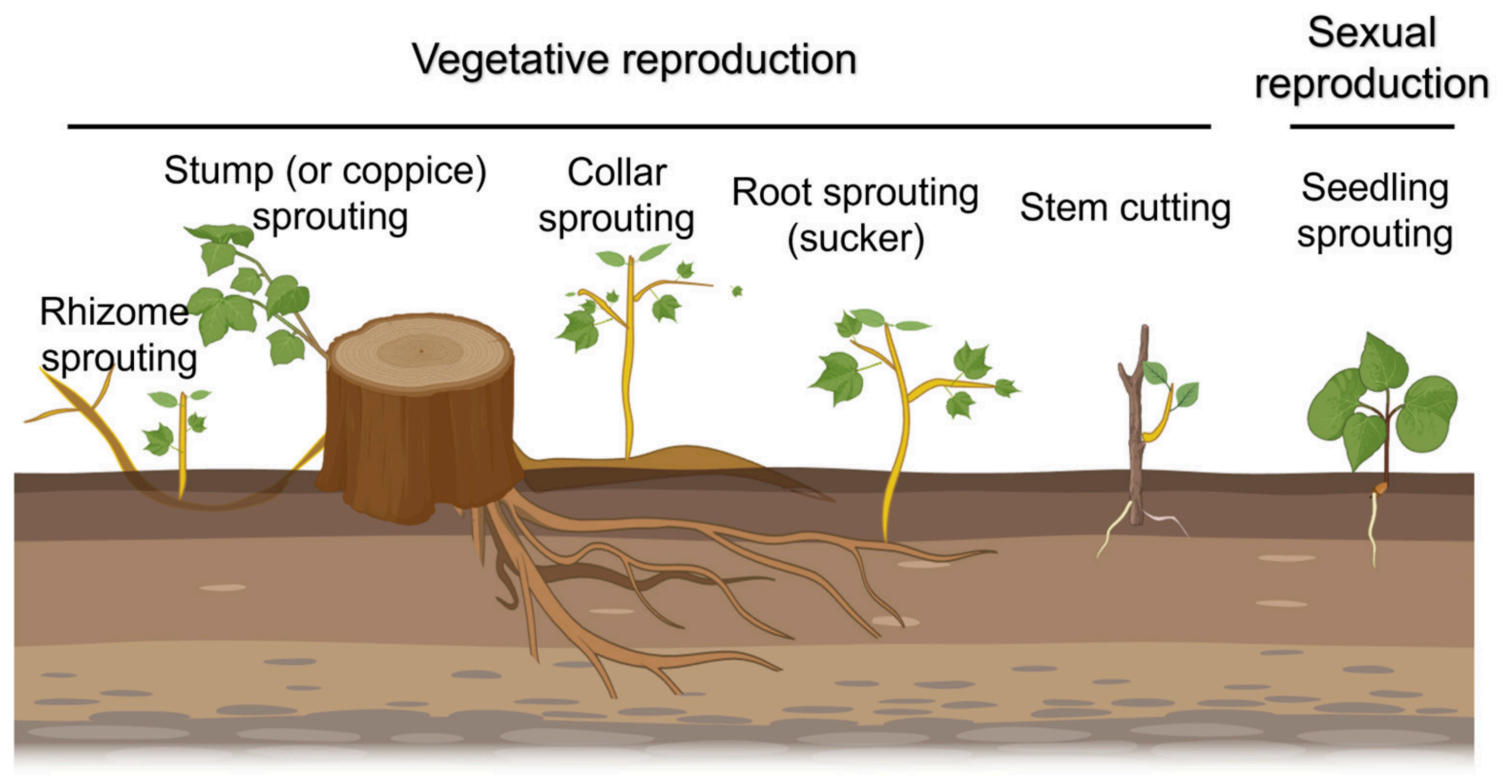
Potential vegetative reproduction in GE trees through sprouting from different anatomical sources. Commercial trees such as aspen and poplar vegetatively reproduce via resprouting mechanisms such as coppicing (or stumps), collars, rhizomes, and root sprouting. Stem cuttings produce clonal plants for commercial purposes by inducing adventitious root (AR) and adventitious shoot (AS) formation in GE tree plantations. This image was rendered on a web-based Biorender platform (https://www.biorender.com/).

According to reviews by Del Tredici and Bond, vegetative tree sprouting represents the emergence of secondary trunks from angiosperms trees, which can lead to the existence of new individuals [61,62]. Four definitive types of vegetative sprouting can generally be identified in trees: collar; special underground stems such as rhizomes; roots; and layered sprouts. Among sprouting types, commercial tree species such as aspen and poplar generally have potential ecological risks of unintended spread through vegetative reproduction via resprouting mechanisms such as coppicing (or stump), collar, rhizome, and root sprouting (Figure 1). The emergence of secondary trunks to form new trees is regulated by the physiological balance between apical dominance and apical control, which is controlled by phytohormone networks [61,63]. Stem cuttings are used to produce clonal plants for commercial purposes by inducing adventitious root formation at the cut stem ends for GE tree plantations (Figure 1). Current field tests of GE poplar and eucalyptus are typically established using clonal propagation via vegetative stem cuttings, which allows continued reproduction without seed harvesting and germination.

It appears that angiosperm trees evolved the capacity for clonal vegetative reproduction without sexual mating by sprouting from spreading roots or detached stems in fields following harvest and in areas subjected to severe environmental damage [64]. Most *Populus* species can clonally reproduce under harsh environmental conditions or disturbance such as fire or flooding. When the aboveground parts of a tree are severely damaged or stumped, an extensive root system can remain intact, which serves as a source to regenerate clones from roots. The ability to survive via vegetative reproduction may enhance the resilience of *Populus* species after environmental disturbance [65].Vegetative reproduction via root sprouting from root and root grafting in poplar are viable methods to recover tree stands for forestry applications.

Root suckering is commonly observed under disturbed field conditions, which may increase its prominence relative to other modes of reproduction. In slash-and-burn agricultural fields, tree reproduction via root sprouting accounts for up to 40% of recovery, which is substantially higher than reproduction through seed germination [66]. The recovery rate of trees regenerated through root sprouting was faster than recolonization by other plant species in logged forests [67,68]. Six years after logging, vegetative reproduction from roots and coppices allowed the recovering of habitats previously occupied by herbaceous plants and shrubs, and restored forestry canopy cover, thereby suppressing the regrowth of competitive understory herbaceous species. These results clearly indicate that vegetative reproduction via root sprouting represents an important potential risk factor for the persistence and spread of GE trees in disturbed wild and plantation ecosystems. Vegetative reproduction ensures that genetically identical clonal spread occurs, unlike for gene flow that occurs through sexual crossing between GE and native trees [30]. Therefore, the persistence of transgenes through vegetative reproduction in trees may be more pronounced than through sexual reproduction via seeds.

Trees have distinctly longer life spans than herbaceous plants and shrubs, and their growth sequentially continues over many years. However, perennial crops, including herbs, shrubs, and vines, also persist for multiple years while reproducing each year. Vegetative reproduction is likewise observed in annual herbaceous crops such as potato, onion, sweet potato, and sugarcane [31]. Thus, increased persistence caused by vegetative reproduction in GE trees may not be a unique phenomenon for trees, although trees live much longer than herbaceous crop plants.

Therefore, when assessing the risks associated with GE trees in field conditions, multiple factors including seedling size, longevity related to fitness and persistence, and transgene dispersal distance must be considered. In particular, risks arising from vegetative reproduction need to be evaluated in relation to competing plant species that coexist with GE trees under field conditions. Consequently, more practical studies on the control of vegetative reproduction and its incorporation into ERA frameworks are required.

## 9. Confinement of Asexual Vegetative Reproduction of GE Trees in the Field

To date, environmental risk mitigation of GE trees in plantations has been conducted by selecting sexually incompatible species surrounding GE trees, planting of border rows or pollen traps, and specific equipment cleaning, transport, and disposal procedures to limit GE tree dispersal in the environment. However, the juvenile phase of GE trees can also lead to spontaneous propagation through sprouting from vegetative organs, potentially causing unexpected spread during the pre-reproductive phase before sexual organ maturation. Furthermore, GE trees can re-establish in post-experiment field conditions by vegetative propagation. Therefore, standardized management measures are required to control risks associated with prolonged living biomass as well as residual plant material after logging, even in cultivated soils.

Since vegetative propagation is practiced in *Populus*, *Eucalyptus*, and some maple species, plantation sites and their perimeters need to be monitored for the spread of vegetative sprouts. To control sprout spread, shade cloths and metal field barriers can be applied to restrict root sucker expansion. Also, herbicide spray and complete burning of plant residues represent a more aggressive way to prevent gene flow through vegetative regeneration. However, these strategies would likely be cost-prohibitive at scale.

**Figure 2:**
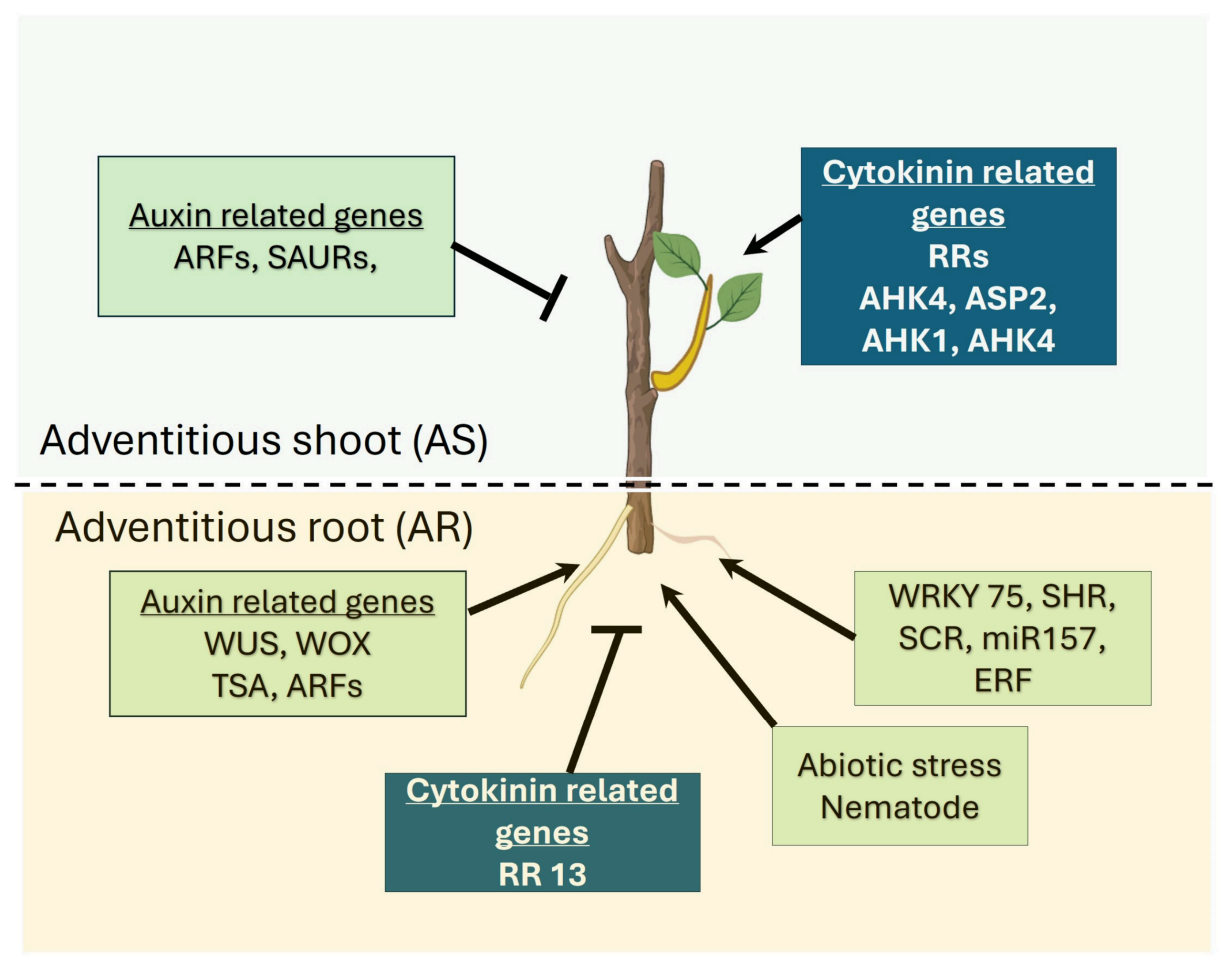
Candidate target genes controlling adventitious shoot and root development during vegetative reproduction in trees. Gene editing or RNA interference targeting these candidates may be an amiable approach to regulate gene spreading risks in GE tree generation (→: activation, ⊥: repression).

Control of the unintended gene flow risk of GE trees has primarily focused on preventing pollination or seed maturation by the impairment of sexual organ or tissue development using additional GE strategies, whose effectiveness has already been evaluated in trees [32,42]. However, even with induced sexual sterility, confinement of field-grown GE trees remains incomplete and unstable owing to the possibility of vegetative reproduction that may result in unexpected spread [32,37]. To that end, male and female sterility strategies could be synergistically enhanced by incorporating control of vegetative reproduction through regulation of genes relating to the initiation and expansion of vegetative reproduction (Figure 2).

Vegetative reproduction of trees can be established from cutting stem ends or root debris remaining in the field (Figure 1). For successful regeneration of individual plants, adventitious stem (AS) or adventitious root (AR) organogenesis is required to establish anchorage and nutrient uptake systems. To date, physiological processes, endogenous hormone dynamics, and nutrient effects during these development stages of trees have been reviewed extensively, especially in poplar. However, many aspects of the molecular mechanisms underlying adventitious tissue establishment remain obscure [69,70,71].

First, gene groups related to root sprouting have been studied using transcriptome analysis of cut roots of white poplar [71]. This study identified three typical stages of AS organogenesis based on anatomical and developmental criteria: primordia initiation, shoot apical meristem (SAM) establishment, and organ differentiation over a six-day period. Physiological experiments conducted under greenhouse conditions revealed a reciprocally antagonistic interaction between auxin for repression and cytokinin for activation in sequential processes during suckering. The expression of phytohormone-related and shoot development-related genes was dependent on sucker developmental stages. During the sucker primordia initiation stages, genes involved in auxin and cytokinin biosynthesis and signaling, along with cell-cycle re-entry-related genes, were upregulated [71].

Nineteen hormone-responsive genes were enriched in the SAM establishment stage, including auxin-responsive genes such as *ARF*s and *SAUR*s, together with cytokinin-response genes *RR9-1*, *RR9-2*, and cytokinin receptor of *AHK4*. In the final organ differentiation stage, common genes related to photosynthesis, photosystem antenna proteins, and cytokinin signaling components and receptors such as *ARF5*, *AHK4*, *APS1*, *ASP2*, *AHK1*, *RR4*, *RR5*, and *RR9* were highly expressed. Therefore, these gene candidates involved in root sucker primordia formation and SAM maintenance may serve as promising targets for genetically engineered trees produced to prevent vegetative reproduction through AS generation (Figure 2).

Second, the successful formation of AR is a critical process for clonal propagation and the survival of GE tree in field conditions, too [70]. Vegetative reproduction through AR formation from suckers or abscised branches generally shows limited potential under natural field conditions. However, this type of reproduction is widely successful in commercial forestry and is commonly applied for GE tree propagation in both laboratory and greenhouse conditions.

Root phenotyping studies of *Populus* as a well-studied tree species indicate that AR performance from stem cuttings is genotype-dependent, implicating that host selection must be considered when applying target genes for confinement of tree transgene flow. AR formation proceeds sequentially through induction, activation, and outgrowth stages, resulting in the development of a new clonal individual. Several quantitative trait loci (QTL) have been associated with AR development. In addition, RNA-seq analysis of stem cuttings at different AR developmental phases as well as fully developed roots has been useful to identify candidate genes associated with distinct stages of AR development. These findings provide a basis for designing control systems to limit vegetative reproduction by impairing AR formation [71,72,73,74,75].

Genes such as *SUR2* and *TSA1* that are involved in auxin (IAA) synthesis during AR formation in *Populus deltoides* and *Populus trichocarpa* pseudo-backcross populations have been identified as important regulators [58]. Most influential regulators of AR formation and elongation are the reciprocal antagonism between auxin and cytokinin, similar to that observed during AS formation. Generally, auxin promotes AR formation in a dose- and stage-dependent manner [76]. Excessive IAA levels inhibited AR formation at later developmental stages.

Other phytohormones including gibberellins, salicylic acid, abscisic acid, brassinosteroids, ethylene, and strigolactones were implicated in AR formation based on physiological analysis. However, their precise functions and underlying mechanisms are still ambiguous. A recent study identified hydrogen peroxide as a mediator of AR and lateral bud formation through the activation of *WRKY75* in poplar [77]. In addition to abiotic stress responses such as cutting, biotic stress is also a potential inducer of vegetative reproduction via AR formation. For example, nematode infection in Arabidopsis resulted in induction of AR in primary roots through the activation of *Wuschel-related homeobox 11 (WOX11)* [75]. Such biotic factors may contribute to unexpected vegetative reproduction under field conditions.

In *Populus* species, *WOX5* and *WOX11/12* are strongly induced during AR regeneration [78], which leads to the control of root development via regulation of D-type cyclin gene [79]. Numerous additional regulators, including *miRNA157*, *ARF17*, *WUS*, *SCARECROW(SCR)*, *SHORT-ROOT(SHR)*, *ERF003*, and *TIR1* have been reviewed as important participants in AR initiation and formation, making them possible targets for controlling vegetative reproduction in *Populus* including cytokinin related RR-13 repressed AR formation [70].

Taken together, many candidate genes involved in both AS and AR formation are primarily associated with auxin and cytokinin signaling pathways (Figure 2). In addition to established strategies for GE tree containment based on controlling pollination and seed dispersal, targeting vegetative reproduction in the field represents a complementary and potentially effective approach to enhance the ecological safety of GE trees in the field. As gene editing is mostly accepted as a safe approach, gene editing targets related to vegetative reproduction may be a promising future approach to control transgene spread in commercial plantation of GE trees. However, it may not yet be a completely reliable approach in the view of environmental concerns. Therefore, the GE trees developed using gene editing should also undergo long-term ERA under field conditions. Through this approach, fitness, development, environmental stress tolerance, and ecological performance associated with the desired transgenic traits can be evaluated and ensured.

## 10. Conclusions

Recent advances in gene editing techniques have led to improvements of GE tree development, which may also serve to decrease public resistance to the commercialization of GE plant products. However, commercial markets still require strong evidence of biosafety for GE products and demand rigorous risk assessments for ensuring biosafety before commercialization.

GE tree usage in forestry and in natural settings must be predicated on satisfactory ERA by field trials. Field tests of several GE trees are currently underway worldwide for commercialization in the near future. Biosafety would include tolerable levels of fitness and invasiveness of GE trees, persistence of transgenes through hybridization and introgression, and controlled vegetative reproduction. For controlling gene flow of GE trees, current efforts through impairment of sexual reproduction of GE trees appear to be effective to minimize transgene dispersal. However, bioconfinement may still be incomplete owing to vegetative reproduction characteristics of some tree taxa in some environments. Therefore, a holistic view for biocontainment is needed to address the sexual and asexual spread of trees over ecological time.

## Data Availability

No new data were created or analyzed in this study. Data sharing is not applicable to this article.
